# Cardiac fibrosis in myocardial infarction—from repair and remodeling to regeneration

**DOI:** 10.1007/s00441-016-2431-9

**Published:** 2016-06-21

**Authors:** Virpi Talman, Heikki Ruskoaho

**Affiliations:** Division of Pharmacology and Pharmacotherapy, Faculty of Pharmacy, University of Helsinki, P.O. Box 56, FI-00014 Helsinki, Finland

**Keywords:** Cardiac fibrosis, Myocardial infarction, Pro-fibrotic signaling, Anti-fibrotic therapy, Cardiac regeneration

## Abstract

Ischemic cell death during a myocardial infarction leads to a multiphase reparative response in which the damaged tissue is replaced with a fibrotic scar produced by fibroblasts and myofibroblasts. This also induces geometrical, biomechanical, and biochemical changes in the uninjured ventricular wall eliciting a reactive remodeling process that includes interstitial and perivascular fibrosis. Although the initial reparative fibrosis is crucial for preventing rupture of the ventricular wall, an exaggerated fibrotic response and reactive fibrosis outside the injured area are detrimental as they lead to progressive impairment of cardiac function and eventually to heart failure. In this review, we summarize current knowledge of the mechanisms of both reparative and reactive cardiac fibrosis in response to myocardial infarction, discuss the potential of inducing cardiac regeneration through direct reprogramming of fibroblasts and myofibroblasts into cardiomyocytes, and review the currently available and potential future therapeutic strategies to inhibit cardiac fibrosis.

**Graphical abstract:**
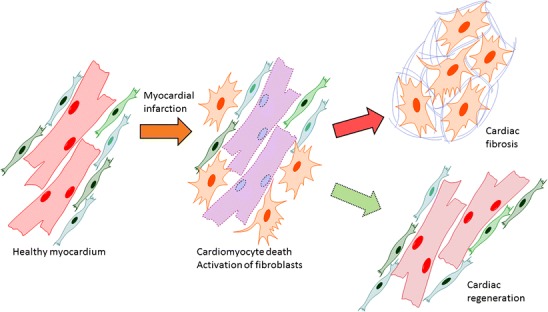
Reparative response following a myocardial infarction. Hypoxia-induced cardiomyocyte death leads to the activation of myofibroblasts and a reparative fibrotic response in the injured area. *Right top* In adult mammals, the fibrotic scar formed at the infarcted area is permanent and promotes reactive fibrosis in the uninjured myocardium. *Right bottom* In teleost fish and newts and in embryonic and neonatal mammals, the initial formation of a fibrotic scar is followed by regeneration of the cardiac muscle tissue. Induction of post-infarction cardiac regeneration in adult mammals is currently the target of intensive research and drug discovery attempts

Reparative response following a myocardial infarction. Hypoxia-induced cardiomyocyte death leads to the activation of myofibroblasts and a reparative fibrotic response in the injured area. *Right top* In adult mammals, the fibrotic scar formed at the infarcted area is permanent and promotes reactive fibrosis in the uninjured myocardium. *Right bottom* In teleost fish and newts and in embryonic and neonatal mammals, the initial formation of a fibrotic scar is followed by regeneration of the cardiac muscle tissue. Induction of post-infarction cardiac regeneration in adult mammals is currently the target of intensive research and drug discovery attempts

## Introduction

Heart failure (HF) is a major public health issue that affects more than 23 million people globally (Bui et al. 2011), and because of the aging population, its prevalence is increasing. Most often, HF is caused by a myocardial infarction (MI). Following an MI, up to 1 billion cardiac cells die in response to ischemia (Laflamme and Murry 2005). The adult mammalian heart has a very limited capacity to regenerate after injury, and the lost cells are replaced by a fibrotic scar. This is followed by remodeling of the surrounding myocardium and eventually leads to impaired cardiac function. The remodeling process includes thickening (hypertrophy) and stiffening (fibrosis) of the left ventricular wall (Sutton and Sharpe 2000).

The fibrotic response after an MI can be classified into two types of fibrosis, namely replacement and reactive fibrosis, both of which are mediated by fibroblasts and myofibroblasts. Replacement fibrosis, i.e. scar formation, is a pivotal process to prevent the rupturing of the ventricular wall after an ischemic insult (van den Borne et al. 2010; Shinde and Frangogiannis 2014). However, the increased mechanical stress post-MI, together with hormonal and paracrine mediators, also induces the expansion of connective tissue in areas remote to the infarction. This reactive fibrosis in the infarct border zone and in the remote uninjured myocardium leads to altered chamber compliance and increased ventricular stiffness thereby compromising cardiac output.

In addition to its effect on cardiac contractility, both the fibrous scar and interstitial fibrosis have been shown to interfere with the normal electrical function of the heart thus predisposing to arrhythmia (for a review, see Francis Stuart et al. 2015). The compact scar may serve as an insulated non-excitable area that anchors re-entrant arrhythmia leading to sustained ventricular tachycardia (Ripplinger et al. 2009). In interstitial fibrosis, the non-conducting fibrillar collagen network between cardiomyocyte sheets might promote re-entrant tachycardia through inducing focal ectopic activity and through slowing or blocking of conduction (Francis Stuart et al. 2015). Additionally, the electronic coupling of myofibroblasts and cardiomyocytes might play a role in fibrosis-induced arrhythmogenesis (Kohl and Gourdie 2014). Not surprisingly, cardiac fibrosis has been identified as an autonomous risk factor in HF: it predisposes HF patients to sudden cardiac death and increases overall mortality independently of the ejection fraction (Gulati et al. 2013).

In this review, we discuss the roles of the various cell types and signaling factors in regulating the repair of infarcted myocardium and in promoting post-infarction pathological reactive fibrosis. We review the potential of inducing cardiac regeneration through the direct reprogramming of fibroblasts and myofibroblasts into cardiomyocytes. We also discuss the therapeutic opportunities for targeting fibroblasts and myofibroblasts in order to restrict reactive fibrosis and to induce cardiac regeneration.

## Fibroblasts and myofibroblasts

**Fig. 1:**
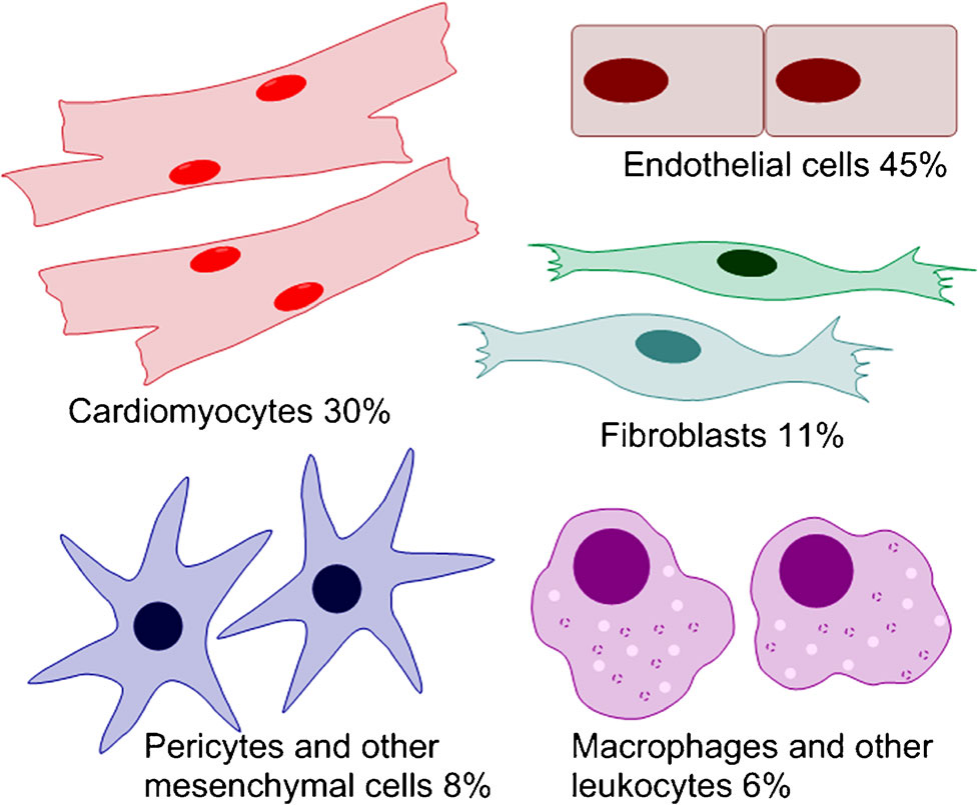
Main cardiac cell types and their relative abundance in adult mouse ventricles. Percentages of various cell types are from Pinto et al. (2015). Notably, the relative abundance of each cell type is likely to be dependent on the species, age, gender, and disease state of the investigated subject. For example, the fibroblast population expands after injury. Additionally, the markers used for cell type identification have a significant effect on the cell percentages

The three main cardiac cell types with regard to cell numbers are cardiomyocytes, endothelial cells, and fibroblasts. Their relative numbers probably depend on the species, age, and gender of the subject. In particular, the percentage of cardiomyocytes varies between infants, young individuals, and adults. Furthermore, the lack of a specific and comprehensive marker for fibroblasts has impeded the precise analysis of the relative abundance of the various cardiac cell types: several putative fibroblast markers have been described, but none of them is unique to fibroblasts, and not all fibroblasts express the suggested marker proteins (see, for example, Souders et al. 2009; Zeisberg and Kalluri 2010; Pinto et al. 2015). According to earlier studies, fibroblasts were considered the most abundant non-myocyte cell type, even outnumbering cardiomyocytes in adult mammalian hearts (Banerjee et al. 2007; Krenning et al. 2010; Zeisberg and Kalluri 2010; Deb and Ubil 2014). A recent report by Pinto et al. (2015), however, challenges this view by showing that endothelial cells and cardiomyocytes are the most abundant cell types in adult murine and human hearts, whereas fibroblasts are the third prevalent cell type in cell numbers (Fig. 1). Even though the proportion of cardiac fibroblasts in normal hearts thus seems to be smaller than previously described, fibroblasts remain a central cell type with regard to post-infarction repair and remodeling. Furthermore, in response to injury, the fibroblast population expands and constitutes the majority of the cells in the infarcted area during the post-MI healing phase.

Cardiac fibroblasts are developmentally of mesenchymal origin, and the majority of them differentiate from epicardium-derived cells during cardiac development (see Zeisberg and Kalluri 2010; Deb and Ubil 2014; Moore-Morris et al. 2016). A subpopulation of fibroblasts mainly located in the interventricular septum and in the valves is derived from the endocardium through the endothelial-to-mesenchymal transition (EndoMT), whereas a small proportion of cardiac fibroblasts, mostly found in the right atrium, is derived from the neural crest (Ali et al. 2014; Moore-Morris et al. 2014, 2016). Some evidence exists that cardiac fibroblasts originate from circulating progenitor stem cells that are recruited into the ventricular myocardium during postnatal life (Visconti and Markwald 2006). Cardiac fibroblasts are distributed throughout the heart as strands and sheets between cardiac muscle fibers. They help to preserve the structural integrity of the heart by maintaining the homeostasis of the extracellular matrix (ECM), which provides a scaffold for all cardiac cells. They also respond to a variety of mechanical, electrical, and biochemical stimuli and are thereby vital for normal cardiac function. For example, cardiac fibroblasts secrete various paracrine factors that regulate the functions of cardiomyocytes, endothelial cells, and immune cells. Furthermore, despite their unexcitable character, cardiac fibroblasts have also been shown to make direct cell-cell interactions with cardiomyocytes through gap-junctional proteins, namely the connexins (Cx40, Cx43, and Cx45), both in vitro and in vivo (Kohl and Gourdie 2014; Ongstad and Kohl 2016). However, the regulation and functional relevance of fibroblast-cardiomyocyte coupling in the heart remain to be elucidated.

After an MI, the loss of architectural integrity exposes fibroblasts to mechanical stress, which together with specific hormones, growth factors, and cytokines induces fibroblast proliferation, migration to the injured area, and transdifferentiation into myofibroblasts (van den Borne et al. 2010). Myofibroblasts are cells that exhibit characteristics of both fibroblasts and smooth muscle cells and are not present in healthy myocardium. The most prominent characteristic of myofibroblasts is their migratory and contracting phenotype, which results from the expression of contractile proteins such as α-smooth muscle actin (α-SMA) and non-muscle myosin. They also exhibit extensive endoplasmic reticulum allowing them to synthesize and secrete large amounts of ECM proteins. Various myofibroblast markers have been described, but they all exhibit significant overlap with other cell types (van den Borne et al. 2010). However, α-SMA staining alone or in combination with a fibroblast marker is a commonly used strategy for the identification of myofibroblasts in cardiac tissue. The protomyofibroblast, an immature form of myofibroblast that exhibits actin stress fibers and mature focal adhesions but that does not express α-SMA, has been described in connection with other tissue injuries. Protomyofibroblasts are, however, still to be identified in infarcted myocardium.

In addition to local cardiac fibroblasts, other cell types can transdifferentiate into myofibroblasts and might contribute to cardiac fibrosis. Myofibroblasts derived from hematopoietic bone-marrow-derived progenitor cells, pericytes, epithelial cells of the epicardium (through the epithelial-to-mesenchymal transition, EMT), and endothelial cells (through EndoMT) have been described in cardiac tissue (Möllmann et al. 2006; Zeisberg et al. 2007; van Amerongen et al. 2008; Russell et al. 2011; Duan et al. 2012; Lajiness and Conway 2014; Davis and Molkentin 2014). However, the roles of myofibroblasts derived from the various cell types and their contribution to cardiac fibrosis are not clear and may depend on the type of cardiac injury. Strong evidence however suggests that the epicardium-derived resident cardiac fibroblasts constitute the primary source of activated fibroblasts or myofibroblasts in the ischemic heart (Ruiz-Villalba et al. 2015) and in pressure-overload-induced fibrosis and remodeling (Ali et al. 2014; Moore-Morris et al. 2014). On the other hand, a recent study by Kramann et al. (2015) has shown that perivascular Gli1^+^ mesenchymal-stem-cell-like cells are key contributors in aortic banding-induced ventricular fibrosis. Upon injury, these cells differentiate into myofibroblasts, and their genetic ablation ameliorates fibrosis and preserves cardiac function. Although their role in MI-induced fibrosis has not been investigated, the finding that a substantial proportion of α-SMA-expressing myofibroblasts is derived from Gli1^+^ progenitors in fibrosis of various solid organs after diverse types of injury suggests that they also play a role in ischemia-induced cardiac injury. Taken together, local cardiac fibroblasts seem to represent the most important source of myofibroblasts in response to cardiac injury, but the contribution of other cell types, such as perivascular stem-cell-like cells, cannot be totally ruled out.

## Cardiac extracellular matrix

The cardiac ECM is composed of structural, matricellular, and adhesion proteins that not only provide a structural framework for cardiomyocytes, but also participate in biochemical signaling and restrict the propagation of electrical activity (for reviews, see Dobaczewski et al. 2012; Klingberg et al. 2013). Type I and III collagens are the primary structural proteins in the cardiac interstitium, and in addition to providing mechanical support by stiffening the myocardial wall, they help in transmitting the mechanical force of contraction (Horn and Trafford 2016). Collagens are synthesized and secreted by fibroblasts and myofibroblasts as collagen precursors and acquire their mature fibrillar form after proteolytic cleavage by collagen proteinases, association with matricellular proteins, and self-assembly to fibrils. Collagens can be further cross-linked enzymatically by lysyl oxidases (LOX) or by a reduction in the response to the formation of advanced glycation end-products (AGEs).

In healthy cardiac tissue, the homeostasis of collagens is tightly regulated through the controlled synthesis of new collagen and the degradation of old collagen fibers. Collagens are degraded by a group of endopeptidases called matrix metalloproteinases (MMPs) whose expression and functions are strictly controlled in order to maintain the homeostasis of ECM degradation and synthesis (for a review, see Lindsey et al. 2016). MMPs also participate in regulating fibrotic signaling. For example, MMP9 cleaves the latent form of transforming growth factor β (TGF-β) in vitro leading to its activation, whereas MMP9 depletion leads to diminished fibrotic signaling and attenuated left ventricular fibrosis in aged mice. The MMPs are inhibited by a family of four endogenous tissue inhibitors of metalloproteinases (TIMPs), all of which are expressed in the myocardium (Vanhoutte and Heymans 2010). By inhibiting the degradation of matrix proteins, TIMPs contribute to ECM expansion, although they have also been suggested to regulate cardiac fibrosis in an MMP-independent fashion through direct functions on fibroblasts and myofibroblasts.

The dimeric glycoprotein fibronectin (FN) is expressed by multiple cell types and regulates adhesion and migration of cells (Klingberg et al. 2013). It consists of homologous repeating domains and can be alternatively spliced to produce a longer protein with inserted extra domain A (EDA) or B (EDB). The EDA-containing FN is up-regulated in infarcted myocardium, exhibits proinflammatory functions, and plays a critical role in promoting the myofibroblast phenotype (Serini et al. 1998). Deletion of the EDA domain has also been shown to prevent pathological remodeling and impairment of cardiac performance after an MI (Arslan et al. 2011).

Matricellular proteins are non-structural proteins of the ECM and play major roles in regulating wound healing and tissue repair. Their expression is dynamically controlled in response to injury. Typically, they bind to the structural ECM proteins and exert their effects by activating cell surface receptors. The central matricellular proteins with established functions in the post-MI healing response include thrombospondins (TSPs), tenascins, periostin, osteopontin, and the CCN family of proteins (for an extensive review, see Frangogiannis 2012). TSPs are a family of five stress-inducible secreted glycoproteins that underlie tissue remodeling. TSP-1 and TSP-4 gene expression is up-regulated in both early and late stages of post-MI remodeling correlating with echocardiographic parameters and natriuretic peptide gene expression and thereby reflecting the degree of remodeling (Mustonen et al. 2008, 2012, 2013). TSP-1 exhibits pro-fibrotic effects through the direct activation of TGF-β but has also been suggested to play a role in preventing the expansion of the infarction (Frangogiannis 2012; Mustonen et al. 2013). The post-MI healing process is impaired in TSP-1 knock-out mice because of defective myofibroblast transdifferentiation and insufficient collagen production. In contrast, the evidence indicates a cardioprotective role for TSP-4: deletion of TSP-4 sensitizes mice to cardiac maladaptation, whereas transgenic mice with inducible cardiac-specific TSP-4 overexpression are protected from myocardial injury (Lynch et al. 2012).

Connective tissue growth factor (CTGF, also known as CCN2), is a key mediator of ECM production under pathological fibrotic conditions (Frangogiannis 2012). It promotes the TGF-β-induced excessive production of ECM, and its expression is rapidly up-regulated in fibroblasts after the exposure of cells to various growth factor stimuli. Mice overexpressing CTGF exhibit pronounced cardiac fibrosis in response to pressure overload. Moreover, monoclonal antibody against CTGF protects from adverse cardiac remodeling and left ventricle dysfunction in mice subjected to pressure overload (Szabo et al. 2014). Tenascin C belongs to the tenascin family of highly conserved glycoproteins (see Frangogiannis 2012). It is not expressed in the healthy adult heart, but in response to MI, its expression is markedly and transiently up-regulated in fibroblasts in the border zone between infarcted and uninjured myocardium. Deletion of tenascin C protects from adverse remodeling and fibrosis of the noninfarcted myocardium suggesting that tenascin C plays an important role in mediating reactive fibrosis (Nishioka et al. 2010).

## Replacement fibrosis after MI: phases and repair vs. remodeling

Necrotic cardiomyocyte death induced by oxygen depletion during an MI evokes a sequence of events that aims at preventing further damage and rupture of the ventricular wall by preserving the remaining cells and by replacing the dead cells. Teleostean fish, newts, and embryonic and neonatal rodents are able fully to regenerate an injured area within the myocardium (Becker et al. 1974; Poss et al. 2002; Porrello et al. 2011). In cardiac regeneration, the injured area is initially replaced by a fibrin clot, followed by replacement first with a temporary collagen-based scar and subsequently with normal myocardial tissue. The complex process involves a tightly controlled inflammatory response, emergence of myofibroblasts, induction of cardiomyocyte proliferation, and neovascularization of the regenerating tissue (Jopling et al. 2010; Kikuchi et al. 2010; Gonzalez-Rosa et al. 2012; Aurora et al. 2014). The regenerative capacity of neonatal rodents is gradually lost during the first week of postnatal life, after which little or no regeneration occurs (Porrello et al. 2011). This is because of the incapability of postnatal cardiomyocytes to re-enter the cell cycle and proliferate. Therefore, in adult mammals, the dead cells are replaced with a permanent collagenous scar instead of new cardiac muscle tissue. The healing process after an MI can be divided into three partially overlapping phases: the inflammatory phase, the proliferative phase, and the maturation phase (Table 1).

**Table 1:** Phases of replacement fibrosis after myocardial infarction in adult mammals (*Ang II* angiotensin II, *CM* cardiomyocyte, *col* collagen, *ECM* extracellular matrix, *ET-1* endothelin-1, *FB* fibroblast, *FGF* fibroblast growth factor, *MFB* myofibroblast, *IL* interleukin, *NFκB* nuclear factor κB, *MMP* matrix metalloproteinase, *PDGF* platelet-derived growth factor, *ROS* reactive oxygen species, *TGF-β* transforming growth factor β, *TLR* toll-like receptor, *TNF* tumor necrosis factor)

Response	Inflammatory phase	Proliferative phase	Maturation phase
Time scale	1–3 (5) days	3 days–weeks	Weeks–months
Tissue–level response	Hypoxia and mechanical stretch Complement activation Clearance of dead cells and matrix fragments	Formation of a collagen-based matrix (scar) Establishment of a microvascular network	Scar maturation: tensile strength ↑ and contraction of the scar
Cell-level response	Necrosis of CMs and other cells in the injured area Infiltration of neutrophils, replacement with macrophages and mononuclear cells	Apoptosis of inflammatory cells FB proliferation, migration and activation Transdifferentiation of FBs and other cell types into MFBs Proliferation and infiltration of endothelial cells	Apoptosis of FBs, MFBs and vascular cells Persistence of MFBs
ECM response	ECM degradation ↑ ECM synthesis ↓ Temporary and highly dynamic matrix comprising of fibrin and fibronectin	Synthesis of structural ECM proteins ↑: collagen (initially col-3), laminin Synthesis of adhesion proteins Synthesis of matricellular proteins	Continued ECM turnover: col-3 ↓ col-1 ↑ Collagen cross-linking Compacted collagen-based scar
Signaling molecules/pathways involved	ROS ↑ Cytokine and chemokine expression ↑ (IL-1β, IL-6, TNF) MMP activity/expression ↑ NFκB, TLR	Expression of inflammatory mediators ↓ Angi-II, ET-1, FGF, PDGF TGF-β1, TGF-β2, IL-10	MMP expression ↑ TGF-β3 lysyl oxidases

The initial inflammatory phase is triggered by massive necrotic cell death in the infarct area (for a review, see Frangogiannis 2014). As interstitial fibroblasts, endothelial cells, and resident cardiac mast cells are more resistant to ischemic injury than are cardiomyocytes, they have been proposed to function as effector cells triggering the post-MI inflammatory reaction (Shinde and Frangogiannis 2014). Cardiac fibroblasts produce MMPs that degrade the ECM allowing cell migration into the injured area. Tissue injury activates innate immune signaling, and secretion of chemokines induces leukocyte infiltration into the injured area (Frangogiannis 2014). The CXC chemokines which have a Glu-Leu-Arg (ELR) signature sequence upstream of the CXC motif (ELR+ CXC chemokines) are known to recruit primarily neutrophils, whereas chemokines from the CC subfamily play a role in recruiting marcophages. These inflammatory cells clear the dead cells and ECM fragments from the infarcted area allowing its repopulation with migrating and proliferating immune cells and, in the later phase, myofibroblasts. Marked increases in the cardiac expression of the proinflammatory cytokines, namely tumor necrosis factor (TNF, formerly known as TNFα), interleukin 1 β (IL-1β), and interleukin 6 (IL-6), have been reported in experimental models of MI (see Frangogiannis 2014). Because of their pleiotropic properties and effects on several cell types, their exact functional roles are, however, not well characterized. IL-1 is known to regulate the fibroblast phenotype and may be responsible for delaying fibroblast conversion to myofibroblasts until the infarction area is cleared and ready for the deposition of new ECM (Saxena et al. 2013). Repression of inflammation during the transition from the inflammatory phase to the proliferative phase is not well characterized but might involve inhibitory molecules (so-called STOP signals) and the activation of pathways that suppress inflammation (Frangogiannis 2014).

As the proinflammatory signaling is suppressed, the number of inflammatory cells decreases through apoptotic cell death, and profibrotic signaling takes over (Frangogiannis 2014). At the beginning of the proliferative phase, fibroblasts become the dominant cell type in the infarct area and adopt a proliferative, migratory, and secretory myofibroblast phenotype (Shinde and Frangogiannis 2014). Infiltration of the injured area with myofibroblasts takes place in all species and experimental models of myocardial injury, regardless of their regenerative capacity. The expression and secretion of ECM proteins by fibroblasts and myofibroblasts start from the infarct border zone and progress toward the core infarct area as the cells migrate along the newly synthesized ECM matrix (van den Borne et al. 2010). Myofibroblasts produce large amounts of interstitial collagens (initially type III and later on, during the infarct healing, type I collagen). Collagen deposition is crucial for increasing tensile strength and preventing ventricular wall rupture. In addition to ECM structural proteins, myofibroblasts secrete increased amounts of FN, particularly EDA-FN, and various matricellular proteins, such as TSPs and tenascin C, which further promote myofibroblast migration and participate in regulating the healing response (Frangogiannis 2012). Furthermore, angiogenic signaling stimulates the proliferation and infiltration of endothelial cells and leads to the establishment of a microvascular network to the infarct area (Jaffer et al. 2006; Deb and Ubil 2014; Frangogiannis 2014); this network is crucial for supplying the myofibroblasts with enough oxygen and nutrients during the repair process.

Following the establishment of a collagen-based matrix at the infarct site, the growth factors and matricellular proteins promoting the survival and activity of myofibroblasts are depleted (van den Borne et al. 2010; Shinde and Frangogiannis 2014). In response, the majority of myofibroblasts are removed from the scarred area, possibly through apoptosis. Moreover, the vascular cells die, and the temporary microvasculature is disintegrated. Whether active inhibitory signaling is involved in suppressing the fibrotic response is unclear. During the maturation phase of MI, collagen turnover by the remaining myofibroblasts continues, and type III collagen is replaced with type I collagen. Type I collagen is further modified by LOX-catalyzed cross-linking. The expression of all four LOX isoforms is increased in the infarct area and in the border zone at 3–7 days post-MI (Gonzalez-Santamaria et al. 2016). This correlates with significant accumulation of mature collagen fibers and extensive remodeling, and LOX inhibition with a pharmacological inhibitor or a neutralizing antibody reduces infarct expansion resulting in improved cardiac function at 28 days post-MI (Gonzalez-Santamaria et al. 2016). Cross-linking of the collagen fibers leads to increased tensile strength and contraction of the scar, which alters the geometry of the chamber and contributes to remodeling in the remote areas of the ventricular wall (van den Borne et al. 2010). In a normal wound healing response, all myofibroblasts are cleared from the scarred area, but in the heart, they have been found to persist in the infarct scar even decades after the insult (Willems et al. 1994). The reason for the continuous myofibroblast presence in the infarct scar is not known but is possibly necessary for the continuous maintenance of the ECM in the continuously contracting environment (van den Borne et al. 2010).

## Reactive fibrosis: remodeling of remote myocardium

Most often it is not the necrotic cardiomyocyte loss during MI that causes heart failure but the subsequent remodeling of the non-infarcted left ventricular wall. In pathological remodeling, the fibroblast-mediated expansion of the ECM is accompanied by the hypertrophic growth of cardiomyocytes as the cells try to compensate for the increased workload by growing in size in order to increase cardiac function and decrease ventricular wall tension (Heineke and Molkentin 2006). The increased thickness caused by cardiomyocyte hypertrophy and stiffness attributable to excessive cross-linked collagen and the tonic contraction of fibrous tissue mediated by myofibroblasts compromise the diastolic function of the heart (Weber et al. 2013). This remodeling process is progressive and eventually leads to the development of heart failure.

The exact mechanisms and regulation of reactive fibrosis are unclear, and systematic studies examining the characteristics of fibroblasts in the non-infarcted myocardium are lacking (Shinde and Frangogiannis 2014). One promoting factor is the increased mechanical stress in the non-infarcted left ventricular wall; this stress also induces the activation of latent TGF-β in the non-infarcted myocardium. In addition, the persisting activated myofibroblasts in the infarct scar continue to secrete pro-fibrotic factors that might traverse to the remote areas of the myocardium inducing activation and proliferation of local fibroblasts and increased collagen deposition in the interstitial compartment (interstitial fibrosis) and in the adventitia of coronary vessels (perivascular fibrosis; Weber et al. 2013). Pro-fibrotic factors initiating and sustaining the reactive fibrotic response are described in the next section.

Whereas interstitial fibrosis stiffens the myocardium and thereby leads to diastolic and systolic dysfunction, reactive fibrosis in the adventitia of the coronary arteries and arterioles (perivascular fibrosis) can cause narrowing of the vessel lumen and has been associated with impaired coronary blood flow (Dai et al. 2012). This might decrease the oxygen supply to the myocardium thereby compromising the survival of cardiomyocytes and predisposing them to ischemic cell death.

## Pro-fibrotic signaling in myocardium

**Fig. 2:**
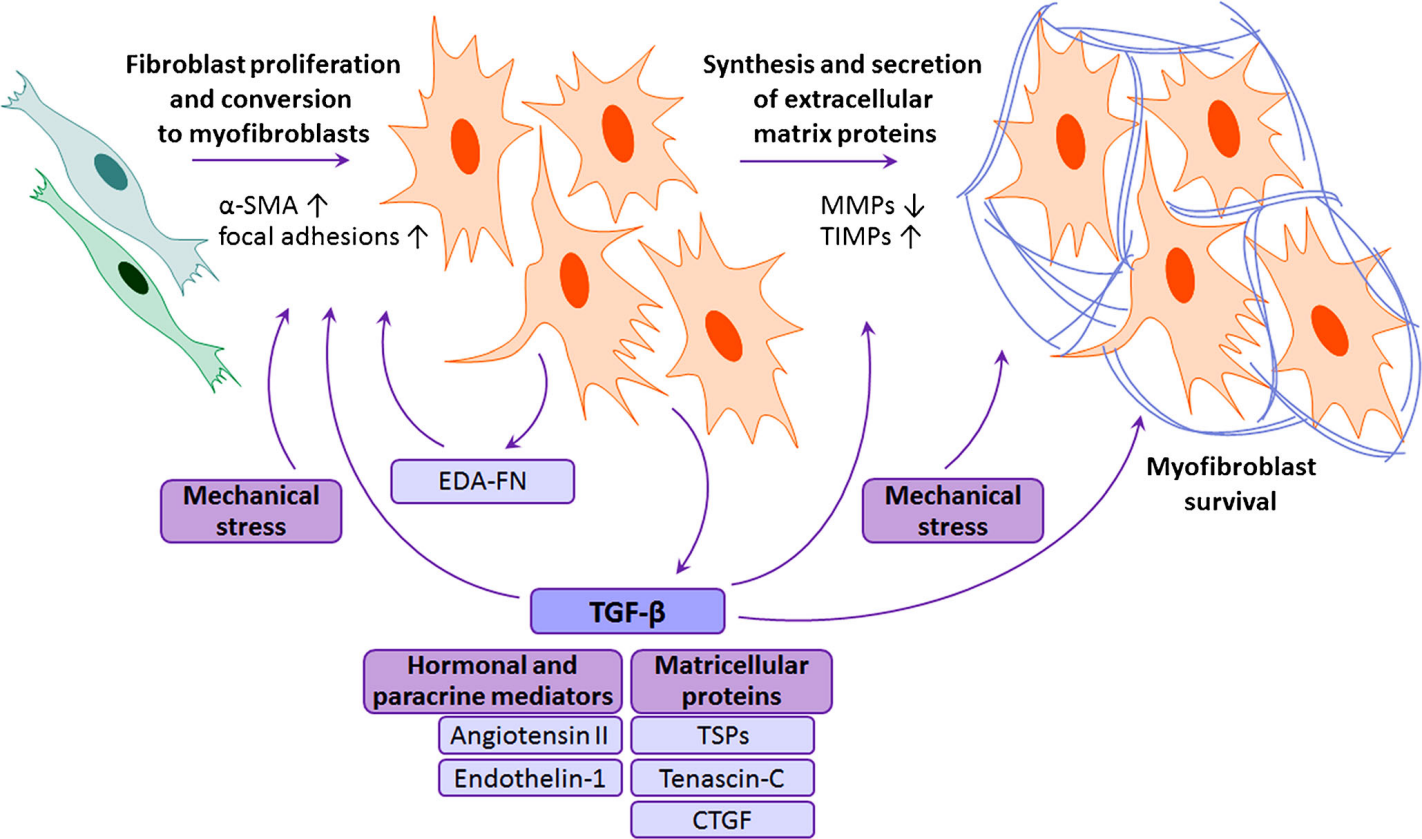
Central pro-fibrotic signaling factors and their effects on fibroblast proliferation, transdifferentiation to myofibroblasts, and extracellular matrix deposition (*α-SMA* α-smooth muscle actin, *CTGF* connective tissue growth factor, *EDA-FN* extra-domain-A-containing fibronectin, *MMPs* matrix metalloproteinases, *TGF-β* transforming growth factor β, *TIMPs* tissue inhibitors of matrix metalloproteinases, *TSPs* thrombospondins)

The function of cardiac fibroblasts and the fibrotic response in the myocardium are regulated by ECM through matricellular proteins (as described above) and through direct ECM-fibroblast connections mediated by transmembrane receptors called integrins (for a review, see Chen et al. 2016). In addition, numerous hormonal, paracrine, and autocrine factors play a critical role in controlling post-MI fibrosis (Fig. 2). TGF-β is probably the best-characterized pro-fibrotic growth factor (Dobaczewski et al. 2011; Kong et al. 2014). Three TGF-β isoforms (1, 2, and 3) exist in mammals, but our knowledge is mainly limited to TGF-β1. In vitro, TGF-β induces myofibroblast transdifferentiation and enhances ECM protein synthesis (Desmouliere et al. 1993). Plenty of evidence also exists for its profibrotic role in vivo as obtained by using both cardiac overexpression and loss-of-function approaches (see Kong et al. 2014). In the healthy heart, TGF-β is present as a latent complex that cannot associate with and activate its receptors but that can be rapidly released and activated in response to reactive oxygen species (ROS) generation, the activation of proteases, mechanical strain, and the induction of matricellular proteins such as TSPs (Buscemi et al. 2011; Frangogiannis 2014). Additionally, TGF-β is synthesized and secreted by platelets, leukocytes, and fibroblasts in the infarcted myocardium (Dobaczewski et al. 2011).

TGF-β1 exerts its effects through binding to its constitutively active tyrosine kinase receptor, namely type II TGF-β receptor (TβRII), at the cell surface. Ligand binding to TβRII recruits the type I receptor (TβRI, also known as ALK5) and induces its transphosphorylation. The intracellular signaling routes include the Smad-dependent regulation of gene expression and the Smad-independent activation of signaling cascades including mitogen-activated protein kinase (MAPK) signaling and signaling through the small GTPase Rho. In particular, signaling through TGF-β-activated kinase (TAK1) and p38 MAPK has been implicated in myofibroblast transdifferentiation, and pharmacological p38 MAPK inhibition is protective against cardiac fibrosis in a rat model of MI (see Lighthouse and Small 2016). Strong evidence also supports an important role for Smad3-dependent TGF-β signaling in the development of post-MI fibrosis; Smad3 null animals have been reported to exhibit less dilative remodeling and attenuated diastolic dysfunction, despite similar infarct sizes (Bujak et al. 2007). This has been attributed to a “hypofunctional” phenotype of infiltrated fibroblasts (increased proliferation accompanied with impaired myofibroblast transdifferentiation and decreased ECM protein deposition; Dobaczewski et al. 2010).

The octapeptide angiotensin II (Ang II) is the central signaling molecule of the renin-angiotensin system (RAS) with regard to cardiac fibrosis. Its immediate in vivo effects include vasoconstriction and increased blood pressure, but it also has direct remodeling-inducing effects on various cardiac cell types (see Leask 2015). At the cellular level, Ang II promotes fibroblast proliferation, myofibroblast transdifferentiation, ECM turnover, and the secretion of proinflammatory cytokines and growth factors. It is expressed and activated by fibroblasts, myofibroblasts, and macrophages in the heart, and by acting on its type I receptor (AT_1_ receptor), it up-regulates the expression of TGF-β and IL-6 in cardiomyocytes, fibroblasts, and myofibroblasts. Both Ang II and TGF-β synthesized and secreted at the infarction site have been suggested to play a role in the development of reactive fibrosis in the non-infarcted myocardium (Weber et al. 2013). They may be able to traverse from the infarcted area to the peri-infarct and to remote areas and might induce fibroblast proliferation and collagen synthesis and secretion in the non-infarcted area. However, no direct evidence of this phenomenon has been presented.

The RAS also promotes fibrosis in an Ang II-independent manner. One key component of the local RAS in the heart is the (pro)renin receptor (PRR; Bader 2010). By binding to PRR, prorenin becomes catalytically active, thus inducing the generation of Ang II. However, renin or prorenin binding to PRR also induces the activation of intracellular signaling that results in the up-regulation of pro-fibrotic genes (Nguyen 2011). In normal rats, PRR gene delivery into the heart induces deleterious myocardial fibrosis associated with the increased expression of various pro-fibrotic genes, such as TGFβ1, CTGF, collagen 1α1, plasminogen activator inhibitor-1, and fibronectin-1, indicating that PRR plays a critical role in hearts undergoing the fibrotic remodeling process (Moilanen et al. 2012). The effects of PRR overexpression are not antagonized by the AT1 receptor antagonist losartan indicating an Ang II-independent mechanism for PRR-mediated myocardial remodeling.

Endothelin-1 (ET-1) is predominantly produced by endothelial cells, but other cardiac cell types (fibroblasts, cardiomyocytes, and macrophages) can also synthesize and secrete it (Rodriguez-Pascual et al. 2014; Leask 2015). ET-1 is a potent pro-fibrotic mediator that seems to act downstream of TGF-β and Ang II, as both of them induce its secretion (Leask 2010; Kong et al. 2014). Similarly to Ang II and TGF-β, ET-1 enhances the proliferation of cardiac fibroblasts and promotes ECM protein synthesis in vitro (Kong et al. 2014). Cardiac-specific overexpression of ET-1 induces fibrosis, and ET-1 antagonism has been shown to reduce fibrosis in an animal model of MI (Oie et al. 2002; Mueller et al. 2011). However, clinical trials with ET receptor antagonists have not shown beneficial effects on cardiac fibrosis or remodeling (Kohan et al. 2012).

Other factors that are known to participate in pro-fibrotic signaling and thereby potentially to promote cardiac fibrosis include ROS, fibroblast growth factor (FGF), and platelet-derived growth factor (PDGF; Leask 2015). PDGF has been suggested to play a role in the proliferation and maturation phases of MI healing: elevated levels of PDGF-A, PDGF-D, and PDGF receptors have been detected in endothelial cells, macrophages, and myofibroblasts in a murine model of MI from post-MI days 3–7 onward (Zhao et al. 2011).

An emerging concept in the regulation of cardiac fibrosis is the involvement of non-coding RNAs. Several microRNAs (miRNAs) that are thought either to promote (miR-21, miR-34, miR-199b, miR-208) or to inhibit (miR-1, miR-26a, miR-29, miR-101, miR-122, miR-133/miR-30, miR-133a, miR-214) cardiac fibrosis have been identified (for reviews, see Thum 2014; Piccoli et al. 2016). Of the anti-fibrotic miRNAs, miR-1 and miR-133 are of particular interest as they have been successfully used for the direct reprogramming of fibroblasts to cardiomyocytes in combination with cardiac transcription factors or miR-208 and miR-499 (see the next section). miR-1 and miR-133 attenuate left ventricular fibrosis in experimental pressure overload (Matkovich et al. 2010; Karakikes et al. 2013). Both of them also play a role in cardiac hypertrophy (Care et al. 2007; Karakikes et al. 2013). Whereas the anti-fibrotic effect of miR-1 has been suggested to be indirect (Thum 2014), miR-133 has been shown directly to suppress collagen expression, both in vitro and in vivo (Shan et al. 2009; Castoldi et al. 2012). Furthermore, the miRNAs of the lethal-7 (Let-7) family have recently been shown to play an important role in post-MI remodeling (Tolonen et al. 2014). Inhibition of Let-7c with an intravenously administered antagomir attenuates myocardial fibrosis and maintains the left ventricular systolic function in mice after ligation of the left anterior descending coronary artery. The Let-7 family of miRNAs also function as suppressors of stem cell pluripotency by regulating the expression of pluripotency genes Oct4 and Sox2 (Roush and Slack 2008). Let-7 inhibition increases the expression of pluripotency genes in cardiac fibroblasts and in the hearts of adult mice, suggesting that the inhibition of Let-7 is a potential approach for inhibiting detrimental post-MI fibrosis (Tolonen et al. 2014). Long non-coding RNAs (lncRNAs) and circular RNAs are additional, more recently described types of non-coding regulatory RNA molecules. However, their roles in cardiac fibrosis are however still mostly unclear (Piccoli et al. 2016).

## Fibroblast reprogramming - harnessing fibrosis to induce regeneration

As the optimal therapeutic goal for post-MI treatment would be to reduce fibrosis and to induce the regeneration of the myocardial tissue through the generation of novel cardiomyocytes, the fibroblasts and myofibroblasts that invade the injured area in order to replace the damaged tissue with a scar represent an attractive target for therapeutic intervention. Indeed, because of the injury-induced fibroblast-myofibroblast transdifferentiation process, these cells might represent a population of plastic cells that can be more easily reprogrammed further into another cell type. The direct reprogramming of fibroblasts/myofibroblasts into induced cardiomyocyte-like cells (iCMs) with the help of cardiac transcription factor overexpression was first reported by Ieda et al. in 2010 (Ieda et al. 2010), and since then, several groups have reported successful cardiac reprogramming with various strategies, both in vitro (Table 2) and in vivo (Table 3; for reviews, see, for example, Srivastava and Berry 2013; Fu and Srivastava 2015; Sahara et al. 2015; Srivastava and Yu 2015).

The majority of in vitro reprogramming studies have exploited the forced overexpression of cardiac transcription factors to induce a direct conversion from fibroblasts to induced cardiomyocytes without passing through a pluripotent or progenitor state (see Table 2 for details and references), whereas some have combined a transient induction by Yamanaka reprogramming factors with subsequent culture in cardiogenic medium to produce iCMs through a progenitor cell stage (Efe et al. 2011; Talkhabi et al. 2015). Furthermore, cardiac reprogramming with the help of miRNAs alone or in combination with transcription factor overexpression has been reported (Nam et al. 2013; Jayawardena et al. 2014; Muraoka and Ieda 2014; Jayawardena et al. 2015; Zhao et al. 2015). The epigenetic reprogramming of fibroblasts into iCMs seems to be stable, as withdrawal of the transcription factors used after 10 days does not affect the morphology or Ca^2+^ oscillations of the resulting iCMs (Addis et al. 2013).

**Table 2:** Conditions used in vitro to induce direct reprogramming of fibroblasts into cardiomyocytes (*BMP4*, bone morphogenetic protein 4, *CDM* chemically-defined medium, *CFs* cardiac fibroblasts, *CPLI* combination of CHIR99021, PD0325901, LIF, and insulin, *CRFV* combination of CHIR99021, RepSox, forskolin, and valproic acid, *cTnT* cardiac troponin T, *EFs* embryonic fibroblasts, *ESC-FBs* embryonic stem cell-derived fibroblasts, *JAK* Janus kinase, *M3-Mef2c* MyoD transactivation domain fused to Mef2c, *PKB* protein kinase B, *SCPF* combination of SB431542, CHIR99021, parnate, and forskolin, *SFs* skin fibroblasts, *TTFs* tail-tip fibroblasts)

Reference	Species	Gene overexpression	miRNAs	Growth factors or small molecules	Readout	Efficiency^a^
Ieda et al. 2010	Mouse	*Gata4, Mef2c, Tbx5*	–	–	cTnT^+^	CFs: 7.5 % TTFs: 4 %
Efe et al. 2011	Mouse	*Transient Oct4, Sox2, Klf4, c-Myc*	–	CDM + BMP4 + JAK-inhibitor JI1	cTnT^+^	EFs: 39 %
Chen et al. 2012	Mouse	*Gata4, Mef2c, Tbx5*	–	–	αMHC-GFP reporter	0 %
Jayawardena et al. 2012	Mouse	*–*	1, 133, 208, 499	–	αMHC-CFP reporter	CFs: 1–5 %
Jayawardena et al. 2012	Mouse	*–*	1, 133, 208, 499	JAK-inhibitor JI1	αMHC-CFP reporter	CFs: 13–27 %
Protze et al. 2012	Mouse	*Tbx5, Mef2c, Myocd*	–	–	cTnT^+^	CFs: 12 %
Song et al. 2012	Mouse	*Gata4, Hand2, Mef2c, Tbx5*	–	–	αMHC-GFP^+^ cTnT^+^	TTFs: 9 %
Addis et al. 2013	Mouse	*Gata4, Mef2c, Tbx5*	–	–	Ca^2+^ activity	EFs: 0.03 %
Addis et al. 2013	Mouse	*Hand2, Nkx2-5, Gata4, Mef2c, Tbx5*	–	–	Ca^2+^ activity	CFs: 4.5 % EFs: 1.6 %
Christoforou et al. 2013	Mouse	*GATA4, TBX5, MEF2C, SRF, MYOCD, SMARCD3, Mesp1*	–	–	MHC-GFP reporter	EFs: 2.4 %
Fu et al. 2013	Human	*GATA4, MEF2C, TBX5, ESRRG, MESP1, MYOCD, ZFPM2*	–	–	αMHC-mCherry + cTnT^+^	ESC-FBs: 13 %
Hirai et al. 2013	Mouse	*Gata4, Hand2, Mef2c, Tbx5*	–	–	cTnT^+^	TTFs: 11 %
Hirai et al. 2013	Mouse	*Gata4, Hand2, M3-Mef2c, Tbx5*	–	–	cTnT^+^	TTFs: 29 %
Nam et al. 2013	Human	*GATA4, HAND2, MYOCD, TBX5*	1, 133		cTnT^+^	HFFs: 22 % AHDFs: 9.5 %
Wada et al. 2013	Human	*GATA4, MEF2C, TBX5, MESP1, MYOCD*	–	–	cTnT^+^	CFs: 5.9 %
Ifkovits et al. 2014	Mouse	*Hand2, Nkx2-5, Gata4, Mef2c, Tbx5*	–	SB431542	Ca^2+^ activity	CFs: 9.3 %
Mathison et al. 2014	Rat	*Triplet Gata4- Mef2c-Tbx5*	–	VEGF	cTnT^+^	CFs: 7.5 %
Muraoka et al. 2014	Mouse	*Gata4, Mef2c, Tbx5*	133	–	cTnT^+^	EFs: 12 %
Nam et al. 2014	Mouse	*Gata4, Hand2, Mef2c, Tbx5*	–	–	α-actinin + cells with sarcomeres	1.2 % of initially plated EFs
Talkhabi et al. 2015	Mouse	Transient *Oct4, Sox2, Klf4, c-Myc*	–	Ascorbic acid, BMP4	MHC^+^	EFs: ≈30 %
Wang et al. 2014	Mouse	*Oct4*	–	Small molecule cocktail SCPF	beating clusters	99 / 10 000 cells plated
Fu et al. 2015	Mouse	*–*	–	Small molecule cocktails CRFV + CPLI	α-actinin^+^	EFs: 15 %
Wang et al. 2015	Mouse	Polycistronic *Mef2c-Gata4-Tbx5*	–	–	cTnT^+^	CFs: 4.9 %
Zhao et al. 2015	Mouse	*Gata4, Hand2, Mef2c, Tbx5*	1, 133	TGF-β inhibitor A83–01	cTnT^+^	EFs: 67 %
Zhou et al. 2015	Mouse	*Gata4, Hand2, Mef2c, Tbx5, Akt/PKB*	–	–	cTnT^+^	EFs: 37 %
Palazzolo et al. 2016	Dog	*GATA4, HAND2, TBX5, MEF2C*	–	–	cTnT^+^	SFs: 12 CFs: 17 %
Zhou et al. 2016	Mouse	Polycistronic *Mef2c-Gata4-Tbx5 + Bmi1* shRNA	–	–	cTnT^+^	CFs: 30 %

^a^Efficiency is expressed as % of cells at the time of analysis, unless otherwise stated

**Table 3:** Conditions used in vivo to induce direct reprogramming of fibroblasts into cardiomyocytes (*CM* cardiomyocyte, *cTnT* cardiac troponin T, *EF* ejection fraction, *FB* fibroblast, *IHC* immunohistochemistry, *VEGF* vascular endothelial growth factor)

Reference	Species	Gene overexpression	miRNAs	Growth factors or small molecules	Readout	Efficiency
Jayawardena et al. 2012	Mouse	*–*	1, 133, 208, 499		Lineage-tracing, cTnT^+^	Evidence of FB-derived CMs
Qian et al. 2012	Mouse	*Gata4, Mef2c, Tbx5*	–		Lineage-tracing, α-actinin^+^	12 % of infected cells
Song et al. 2012	Mouse	*Gata4, Hand2, Mef2c, Tbx5*	–		Lineage-tracing, cTnT^+^	6.5 % of CMs in injured area
Mathison et al. 2014	Rat	*Triplet Gata4- Mef2c-Tbx5*	–	VEGF	IHC, EF	Fibrosis ↓, EF ↑
Jayawardena et al. 2015	Mouse	*–*	1, 133, 208, 499		Lineage-tracing, cTnT^+^	12 % of CMs in peri-infarct area, function ↑
Ma et al. 2015	Mouse	Polycistronic *Mef2c-Gata4-Tbx5*	–		Lineage-tracing, α-actinin^+^	Greater reprogramming efficiency than with individual *Mef2c, Gata4, and Tbx5* vectors, fibrosis ↓

Direct comparison of reprogramming efficiency between the different approaches is difficult because of differences in the experimental setup and the outcome measures used for cardiomyocyte classification. In particular, the time point and the criteria used for classifying cells as iCMs have a dramatic effect on the reported reprogramming efficiency. Classification based on cardiac protein expression (cardiac troponin T [cTnT] or cardiac α-actinin) results in significantly higher reported efficiency than classification by using a functional measure such as Ca^2+^ activity combined with a cardiac-specific reporter or than classification based on the presence of sarcomeres. Nevertheless, certain conclusions with regard to reprogramming efficiency can be drawn from the plethora of studies published. When Ca^2+^ activity was used as the outcome, the combination of *Hand2*, *Nkx2.5*, *Gata4*, *Mef2c*, and *Tbx5* was found to be >50-fold more efficient than the three-factor combination of *Gata4*, *Mef2c*, and *Tbx5* (Addis et al. 2013), suggesting that additional transcription factors, although not necessary for the expression of cardiac proteins such as cTnT, might be important in the maturation of iCMs to functional cardiomyocytes. The highest reprogramming efficiency reported, with cTnT^+^ cells used as a measure of iCMs, is a remarkable 67 % (Zhao et al. 2015). This was achieved by the overexpression of four cardiac transcription factors (*Gata4*, *Hand2*, *Mef2c*, *Tbx5*) and two miRNAs (miR-1, miR-133) combined with the pharmacological inhibition of TGF-β. Inhibition of fibrotic signaling with TGF-β or Rho-associated kinase (ROCK) inhibitors improves reprogramming efficiency, as does the overexpression of Akt/protein kinase B (Ifkovits et al. 2014; Zhao et al. 2015; Zhou et al. 2015).

Most of the in vitro studies have been carried out with primary fibroblasts isolated from mice, but successful cardiac reprogramming has also been reported with primary fibroblasts isolated from rats, dogs, and humans (Table 2). Human fibroblasts have proven to be more resistant to reprogramming than murine fibroblasts: the successful cardiac reprogramming of human fibroblasts into iCMs requires more transcription factors than reprogramming of murine fibroblasts, and the process is slower and less efficient with human cells compared with murine cells. The origins of the fibroblasts also affect reprogramming efficiency, and cardiac fibroblasts are more easily converted to iCMs than other fibroblasts (Ieda et al. 2010; Addis et al. 2013; Palazzolo et al. 2016). This is in agreement with the report showing that cardiac fibroblasts express a number of cardiogenic genes such as transcription factors *Gata4*, *Tbx20*, *Tbx5*, *Nkx2-5*, *Hand2*, and *Mef2c* at significantly higher levels than tail fibroblasts (Furtado et al. 2014). At least a subpopulation of cardiac fibroblasts thus seems to be “primed” for reprogramming.

The reprogrammed iCMs consist of all three major CM subtypes: pacemaker, atrial, and ventricular (Nam et al. 2014). However, the phenotype of iCMs produced by direct reprogramming in vitro resembles that of immature cardiomyocytes with spontaneous Ca^2+^ oscillations and contractions (Sahara et al. 2015). This is consistent with the immature phenotype of cardiomyocytes derived from embryonic stem cells (ESCs) or induced pluripotent stem cells (iPSCs; Gherghiceanu et al. 2011). With regard to clinical applications, an immature phenotype can be considered to increase the risk of arrhythmia, and therefore strategies to promote the maturation of iCMs are needed. The biomechanical and biochemical environment in the myocardium might, however, promote the maturation of iCMs, thereby reducing the risk of proarrhythmia. Most in vivo cardiac reprogramming studies have utilized lineage tracing to demonstrate the origin of in vivo reprogrammed iCMs and a strategy of injecting viral vectors for cardiac transcription factors or miRNAs immediately after the induction of MI with coronary artery ligation (see Table 3 for details and references). Although the numbers of reprogrammed cells detected in the injured area or in the border zone have been quite modest, the iCMs generated in vivo exhibit morphology resembling mature cardiomyocytes and seem to make connections to endogenous cardiomyocytes (Song et al. 2012; Qian et al. 2012; Ma et al. 2015). The environmental clues present in the heart and the epigenetic state of cardiac fibroblasts thus indeed seem to support the maturation and proper electrical coupling of iCMs. Furthermore, the functional improvements observed in response to in vivo reprogramming after MI are more substantial than expected taking into account the relatively modest number of iCMs generated. However, this is not surprising, as intramyocardial *Gata4* gene transfer has been shown to significantly reduce infarct size and improve ejection fraction in a rat model of MI (Rysä et al. 2010). The cardioprotective mechanisms of Gata4 overexpression include the induction of myocardial angiogenesis, the inhibition of apoptosis, and the recruitment of c-Kit^+^ cardiac progenitor cells.

As an alternative approach, recent reports describe three different strategies for reprogramming fibroblasts into induced cardiovascular progenitor cells (iCPCs) in vitro (Table 4). When grown under cell culture conditions that favor cardiomyocyte generation, these iCPCs differentiate into cardiomyocytes (Pratico et al. 2015; Lalit et al. 2016; Zhang et al. 2016). The iCPCs also differentiate into cardiomyocytes, endothelial cells, and vascular smooth muscle cells in vivo after cell transplantation to infarcted myocardium (Lalit et al. 2016; Zhang et al. 2016). The advantage of reprogramming fibroblasts to progenitor cells rather than directly to iCMs lies in the ability of iCPCs to proliferate allowing the expansion of the cell population before differentiation into iCMs (Lalit et al. 2016; Zhang et al. 2016). Whether reprogramming to iCPCs can be achieved in vivo and how the proliferation and differentiation can be controlled remain to be investigated.

**Table 4:** Conditions used in vitro to induce direct reprogramming of fibroblasts into cardiac progenitor cells (*BIO* 6-bromoindirubin-30-oxime, *LIF* leukemia inhibitory factor, *Cxcr4* C-X-C chemokine receptor type 4; *Flk1* fetal liver kinase 1 (also known as kinase insert domain receptor, KDR), *Isl1* ISL LIM homeobox 1, *PDGFRα* platelet-derived growth factor receptor α, *BACS* combination of bone morphogenetic protein 4 (BMP4), activin A, CHIR99021, and SU5042, *5-AZ* 5-azacytidine, *AA* ascorbic acid, *BMP4* bone morphogenetic protein 4, *FGF* fibroblast growth factor, *VEGF* vascular endothelial growth factor)

Reference	Species	Gene overexpression	Growth factors or small molecules	Progenitor characterization	Expansion	Differentiation
Pratico et al. 2015	Human	*GATA4, MEF2C, TBX5, HAND2*	–	c-kit^+^, Isl1^+^, Nkx2-5^+^	–	5-AZ, followed by AA + TGF-β
Lalit et al. 2016	Mouse	*Mesp1, Tbx5, Gata4, Nkx2-5, Baf60c*	BIO, LIF	Nkx2-5-eYFP reporter, Cxcr4^+^	BIO + LIF	Wnt inhibitor IWP-4, BMP4, VEGF, FGF
Zhang et al. 2016	Mouse	*Transient Oct4, Sox2, Klf4, c-Myc*	JAK-inhibitor JI1 + CHIR99021	Flk1^+^, PDGFRα^+^, Isl1^+^, Nkx2-5^+^	BACS	Wnt inhibitor IWP-2

## Effect of current HF drugs on fibrosis

Current HF treatment recommendations are based on RAS inhibition and β adrenergic receptor antagonists, supplemented with mineralocorticoid/aldosterone receptor antagonists, ivabradine, and/or digoxin as necessary (McMurray et al. 2012). In cases with a diagnosis or a high risk of coronary artery disease, cholesterol-lowering drugs are included in the regimen. Despite advances in therapy, the mortality rates for HF are higher than those for many cancers: 40–60 % of patients die within 5 years of diagnosis (see Bui et al. 2011). Although none of the presently available drugs are able to reverse post-infarction remodeling, some have been shown to exhibit anti-fibrotic properties, both in vitro and in vivo, and their clinical efficacy in the treatment of HF may therefore be partly attributable to the inhibition of pathological remodeling.

Because of the well-established role of Ang II in promoting cardiac fibrosis through the AT_1_ receptor-mediated up-regulation of TGF-β1 expression, angiotensin-converting enzyme 1 (ACE1) inhibitors and AT1 receptor blockers (ARBs) unsurprisingly inhibit cardiac remodeling and fibrosis in various experimental models (reviewed in Rosenkranz 2004; Porter and Turner 2009; Weber et al. 2013). In addition to inhibiting the AT_1_ receptor-mediated TGF-β up-regulation, ARBs have been shown to up-regulate the expression of another ACE isoform, ACE2, which hydrolyses angiotensin II into angiotensin [1–7] (see Weber et al. 2013). Signaling through the ACE2—angiotensin [1–7]—Mas receptor axis is cardioprotective, and ARBs have thus been suggested to have additional benefits over ACE inhibitors (Weber et al. 2013). However, early treatment with ARB losartan has also been shown to aggravate cardiac remodeling in a rat model of MI by inducing apoptosis and fibrosis in the peri-infarct area (Serpi et al. 2009). The timing of ARB treatment may thus be critical in order to achieve optimal results.

In contrast to cardiomyocytes with predominant β_1_ adrenergic receptor-mediated signaling, cardiac fibroblasts express mainly β_2_ adrenergic receptors, and β_1_ receptor-mediated signaling plays only a minor role (Porter and Turner 2009; Aranguiz-Urroz et al. 2011; Carter et al. 2014). Stimulation of β_2_ receptors in cardiac fibroblasts has been linked to the increased proliferation of cardiac fibroblasts and the up-regulation of IL-6, and these effects can be blocked with non-selective or β_2_ receptor-selective antagonists, but not with antagonists selective for β_1_ receptors, suggesting that the inhibition of β_2_ receptors is beneficial in reducing fibrosis (see Porter and Turner 2009). However, the effects of IL-6 down-regulation on cardiac remodeling have not been elucidated, and temporal control might be critical for a beneficial effect (see Frangogiannis 2014). The β receptor blockers most frequently prescribed for secondary prevention after MI are β_1_-selective, which has been hypothesized to overlook the potential benefits of blocking β_2_ receptor-mediated signaling in cardiac fibroblasts (Porter and Turner 2009).

Inhibitors of 3-hydroxy-3-methylglutaryl coenzyme A (HMG-CoA) reductase, namely statins, are effective and widely used for both primary and secondary prevention of ischemic cardiovascular events because of their cholesterol-lowering effects. Additionally, increasing evidence suggests that they exhibit anti-remodeling properties, which contribute to their beneficial clinical effects. Under in vitro conditions, statins have been shown to directly inhibit cardiac fibroblast proliferation and migration, fibroblast-myofibroblast transdifferentiation, and ECM turnover, all of which are expected to confer beneficial effects in the myocardial remodeling process (for a review, see Porter and Turner 2009). Statins have also been described to exhibit anti-fibrotic effects in vivo, for example, in animal models of myocardial infarction (Sun et al. 2015; Hayashidani et al. 2002) and metabolic syndrome (Hermida et al. 2013). All in all, statins and other cardiovascular drugs currently in use are, however, not efficient enough in blocking the progression of pathological fibrosis and remodeling, and therefore, new and more efficient anti-fibrotic drugs are needed.

## Concluding remarks and future prospects

ECM homeostasis in the myocardium is essential for normal cardiac function. An efficient reparative scarring process after an MI is also of critical importance for maintaining the structural integrity of the ventricular wall. However, the progressive reactive fibrosis elicited by biomechanical and biochemical changes in the non-infarcted myocardium after an ischemic injury plays a major role in the development of HF. Cardiac fibroblasts and myofibroblasts therefore represent attractive cellular targets for the development of treatments aimed at inhibiting pathological post-infarction remodeling. However, such therapies should specifically inhibit the reactive fibrosis without interfering with the initial reparative scarring process.

In order to stop the reactive fibrosis that contributes to the progression of HF, two strategies can be taken: the inhibition of pro-fibrotic signaling and the activation of anti-fibrotic pathways. As TGF-β plays a central role in promoting fibroblast proliferation, myofibroblast transdifferentiation, collagen deposition, and myofibroblast survival, the inhibition of TGF-β signaling is a promising approach for inhibiting fibrosis. However, in order not to interfere with the scar formation at the site of the injury, TGF-β inhibition should be temporally controlled and initiated only in the post-healing phase after MI. This is supported by the in vitro observation that the inhibition of TGF-β signaling by the overexpression of c-Ski induces the reversal of the myofibroblast phenotype (Cunnington et al. 2011), suggesting that TGF-β inhibition can be used to convert myofibroblasts back to quiescent fibroblasts once the scar is formed. Of note, the inhibition of TGF-β has been linked to aortic aneurysm progression and complications in mice (Wang et al. 2010), emphasizing that the approach is not risk-free. In addition, targeting EDA-FN might provide a means to selectively inhibit reactive fibrosis: EDA-FN knockout mice exhibit reduced reactive fibrosis in the remote non-infarcted myocardium, whereas the level of reparative fibrosis is unaffected (Arslan et al. 2011). Moreover, PRR represents an interesting drug target, and PRR blockers could be combined with ARBs to allow more complete myocardial protection and to prevent the deleterious Ang-II-independent actions of renin that are not inhibited by renin inhibitors (Moilanen et al. 2012). Interesting observations also include the anti-fibrotic effects of neuregulin 1 (NRG1), a growth factor that plays a role in cardiac development and also mediates cardiac regeneration (Kim et al. 2012; Galindo et al. 2014; Harvey et al. 2016). In a swine model of MI, intravenous NRG1 treatment initiated at 1 week post-infarction suppressed fibrosis and improved cardiac function (Galindo et al. 2014). In vitro studies with murine and rat primary cardiac fibroblasts suggest that the anti-fibrotic mechanism of NRG1 is mediated through inhibition of TGF-β signaling and myofibroblast transdifferentiation. Other putative therapeutic strategies to inhibit pro-fibrotic signaling include LOX inhibition, Wnt inhibition, and histone deacetylase inhibition (Hermans et al. 2012; Schuetze et al. 2014; Gonzalez-Santamaria et al. 2016).

The second strategy to limit reactive post-MI fibrosis by activating anti-fibrotic signaling pathways has gained less attention, and the signaling pathways that restrict excessive fibrosis in physiological homeostasis represent an insufficiently investigated area. Natriuretic peptide A (NPPA, ANP) and B (NPPB, BNP) have emerged as important candidates for the development of therapeutic agents for heart failure (Lee and Burnett 2007). Their secretion is markedly up-regulated in HF, and they exhibit important autocrine, paracrine, and endocrine cardioprotective and anti-remodeling activities that are mediated through the guanylyl cyclase-A (GC-A) receptor and the activation of cyclic guanosine monophosphate (cGMP) in target cells (Ruskoaho 1992; Lee and Burnett 2007). In particular, strong evidence supports an anti-fibrotic role for BNP. In cultured fibroblasts, BNP decreases collagen synthesis and up-regulates MMP expression (Tsuruda et al. 2002). Mice lacking the BNP gene have normal-sized hearts but increased ventricular fibrosis (Tamura et al. 2000). Furthermore, local intramyocardial BNP gene delivery improves cardiac function and attenuates post-MI and Ang II-induced fibrosis and adverse remodeling (Moilanen et al. 2011). The enhancement of BNP-mediated effects in the heart would thus be an attractive strategy to inhibit cardiac fibrosis. Another approach for enhancing anti-fibrotic signaling through activating cGMP-mediated pathways is by the inhibition of cGMP-degrading enzymes, the phosphodiesterases (PDEs). PDE5 inhibitors are widely used for erectile dysfunction, and more recent evidence highlights their additional beneficial effects, including the inhibition of fibrosis, in the heart (Kass 2012; Gong et al. 2014; Corinaldesi et al. 2016).

More ambitious is the aim of reversing reparative fibrosis at the infarct site to induce regeneration of the cardiac muscle. The plasticity of cardiac fibroblasts and myofibroblasts and their abundance in the injured area make them a suitable starting cell population for the generation of de novo cardiomyocytes to repair the injury. The success of reprogramming fibroblasts directly into cardiomyocyte-like cells both in vitro and in vivo highlight the potential of this approach for cardiac repair and regeneration. Direct reprogramming would circumvent the need for the cell transplantation that is required for stem cell therapy or approaches involving iPSC-derived cardiomyocytes. Additionally, direct reprogramming would circumvent the risk of potential teratogenicity, which remains a concern with strategies utilizing pluripotent cells and the clinical use of iPSC-derived cardiomyocytes. However, safety issues related to genetic modifications and viral vectors need to be resolved or small-molecule pharmacological agents have to be discovered in order to develop a safe strategy for direct reprogramming in a clinical setting.

**Fig. 3:**
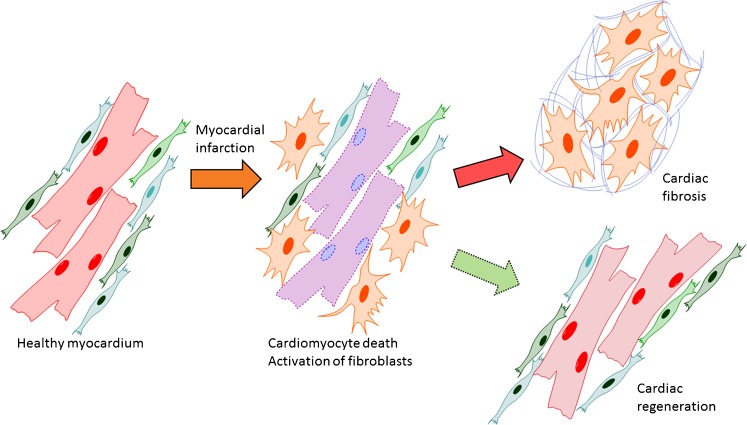
Reparative response following a myocardial infarction. Hypoxia-induced cardiomyocyte death leads to the activation of myofibroblasts and a reparative fibrotic response in the injured area. *Right top* In adult mammals, the fibrotic scar formed at the infarcted area is permanent and promotes reactive fibrosis in the uninjured myocardium. *Right bottom* In teleost fish and newts and in embryonic and neonatal mammals, the initial formation of a fibrotic scar is followed by regeneration of the cardiac muscle tissue. Induction of post-infarction cardiac regeneration in adult mammals is currently the target of intensive research and drug discovery attempts

An ideal therapy for MI-induced cardiac injury would combine the inhibition of reactive fibrosis (and other remodeling processes) in non-infarcted areas with the induction of the regeneration of the infarcted myocardium (Fig. 3), for example, by direct reprogramming of fibroblasts to cardiomyocytes. A more detailed understanding of the gene programmes, signaling cascades, and cellular metabolic routes deciding between regeneration in neonatal rodents or scarring and remodeling in adults is, however, critical for the development of such therapeutics. A strong candidate to be included in such a treatment strategy would be the inhibition of TGF-β signaling, as it restricts adverse fibrotic remodeling and enhances cardiac reprogramming efficiency when combined with cardiac transcription factor overexpression. Moreover, the transcription factors that are central in cardiac development and have been used in reprogramming fibroblasts to cardiomyocytes also participate in mediating pathological adaptation in the heart (Pikkarainen et al. 2004; Clowes et al. 2014). As more information concerning the structures and molecular interactions of these factors is revealed (Luna-Zurita et al. 2016), they are also expected to attract the attention of drug developers. Furthermore, our rapidly expanding knowledge of the significance of non-coding RNAs in controlling cardiac physiology and pathophysiology will possibly bring forward novel approaches for the treatment of cardiac fibrosis.
